# Quality of life, work ability and oral health among patients with chronic liver diseases

**DOI:** 10.4317/medoral.22918

**Published:** 2019-05

**Authors:** Inácio Aguiar, Liliane Lins-Kusterer, Larissa-Souza-Santos Souza, Raymundo Paraná, Jorge Bastos, Fernando-Martins Carvalho

**Affiliations:** 1 Odontólogo, Mestre em Medicina e Saúde, Universidade Federal da Bahia, Salvador, Bahia, Brasil; 2 Associate Professor, Doutora em Patologia Humana, Departmento de Medicina Preventiva e Social, Universidade Federal da Bahia, Salvador, Bahia, Brasil; 3 Estudante de Odontologia, Escola de Odontologia, Universidade Federal da Bahia, Salvador, Bahia, Brasil; 4 Professor Titular, Departmento de Medicina, Universidade Federal da Bahia, Salvador, Bahia, Brasil; 5 Professor Titular, Departmento de Medicina Preventiva e Social, Universidade Federal da Bahia, Salvador, Bahia, Brasil

## Abstract

**Background:**

This study aimed to explore the associations between health-related quality of life and work ability with the oral health status of patients with chronic liver disease.

**Material and Methods:**

A cross-sectional study included 150 patients with chronic liver disease, consecutively seen at University Hospital, Salvador, Brazil. Oral health was evaluated by the Decayed, Missing, and Filled Teeth (DMFT) index and by the presence of gingivitis and periodontitis. Salivary flow was “reduced” when <1.0 mL/min. Health-related quality of life was evaluated by using the 36-Item Short Form Health Survey questionnaire (SF-36); work ability was evaluated by the Work Ability Index questionnaire.

**Results:**

All health-related quality of life indicators were systematically lower among the 99 patients with reduced salivary flow than among the 51 patients with normal salivary flow. Physical Functioning, Role-Physical, and Physical Component Summary scores were strongly correlated (*P*< 0.005 or less) with the number of Missing Teeth and with DMFT index. Reduced salivary flow was associated (*P*< 0.05) with poor work ability. Patients with poor or moderate work ability presented higher (*P*< 0.001) means of the DMFT index than those with good or excellent work ability.

**Conclusions:**

Patients with chronic liver disease who present poor oral health presented low health-related quality of life and poor work ability. These findings reinforce the need of these patients for specialized stomatological care.

** Key words:**Xerostomia, dental health surveys, hepatitis, alcoholic liver disease.

## Introduction

There is increasing concern about the biological, psychological, sociodemographic, clinical and therapeutic factors that may influence the health-related quality of life (HRQOL) of patients with chronic liver diseases (1). Poor HRQOL in such patients has been associated with depression (2) and cirrhosis complications, such as hepatic encephalopathy, ascites, spontaneous bacterial peritonitis, and haemorrhage due to oesophageal varices (3,4).

The compartmentalized development of scientific knowledge has segregated the mouth from the rest of the body. Further, oral health is hardly conceived as part of general health, perhaps because its social and material determinants are not adequately considered (5).

Several factors are associated with poor oral health, such as older age, low levels of education, low income, smoking, drinking, systemic diseases, and medications. Usually, oral health has been evaluated by using clinical criteria that are inadequate to measure the real impact of oral diseases on patient’s life. The quality of cross-cultural adaptation of most oral health-related quality of life instruments has been criticized (6).

Oral manifestations in patients with chronic liver disease have been well described. These patients frequently present reduced salivary flow (7), making them more susceptible to the onset of periodontal diseases and caries. By their turn, such oral manifestations can contribute to the occurrence and worsening of severe complications, such as hepatic encephalopathy (8) and pyogenic liver abscess (9). A systematic review (10) found few published studies about the association between periodontal disease and liver cirrhosis. Most of these studies had cross-sectional design. Only one cohort study reported that mortality was lower among patients who underwent dental treatment versus those who were not treated (7).

To the best of our knowledge, only one preliminary study has reported the association between decreased work ability and oral disease in patients with chronic liver disease (11) 

This study aimed to explore the associations between health-related quality of life and work ability with the oral health status of patients with chronic liver disease.

## Material and Methods

-Study design and patient selection

A cross-sectional study with outpatients with chronic liver disease, aged 18 years or over, of both sexes, consecutively seen at the Hepatology Unit of the University Hospital, Federal University of Bahia, Brazil, between August 2015 and June 2016. Patients should have a MELD score (12) lower than 15. Patients unable to communicate or who had difficulty understanding the study questionnaires were excluded from the study.

-Data collection

Demographic and clinical information were collected from each patient by using a specific form, by only one dental doctor.

Health-related quality of life (HRQOL) was taken as a dependent variable, evaluated by the validated Portuguese version of the 36-Item Short Form Health Survey (SF-36) (13), as recommended by QualityMetric Incorporated (14). The SF-36 has been used to evaluate the HRQOL of people all over the world, including patients with chronic diseases (15). The 36-question form refers to the previous 4-week period. These questions can be used to build eight domains - physical functioning, role limitations due to physical problems, bodily pain, general health perceptions, vitality, social functioning, role limitations due to emotional problems, and mental health, which can be aggregated to a physical component summary and a mental component summary. The raw score of these measurements varies from 0 to 100, where 100 represents the best HRQOL. SF-36 scores were normalised, assuming a mean of 50 and a standard deviation of 10, taking the general population of the USA as a standard. A normalised score below 50 should therefore be interpreted as below the mean for the population of the USA (14). This study was licensed by QualityMetric Health OutcomesTM under number QM025905.

Work ability was also considered to be a dependent variable, measured by the Work Ability Index (16) questionnaire, using a version validated for Brazil (17,18). This instrument is based on self-assessment reports which measure work ability. The raw Work Ability Index score can vary from 7 to 49; but, for the purposes of this study, it was stratified into four categories, as recommended by its developers: 7-27 poor; 28-36 moderate; 37-43 good and 44-49 excellent (16).

The oral health status evaluation was made by the same dental doctor, following criteria recommended by the World Health Organization 17 and the European Association of Dental Public Health (19,20). The DMFT index and its components (number of decayed/missed/filled teeth) were determined. Periodontal disease was ascertained by measuring clinical attachment loss, probing pocket depth, tooth mobility, and panoramic radiography to assess intraosseous lesions. Stimulated saliva was collected in the morning, two hours after breakfast. Participants were asked to sit in a chair while saliva was stimulated by salivary mechanical stimulation using mechanical sialogogue for 2 min. Participants have expectorated the accumulated saliva into a graduated sterile tube. After 2 min, the amount of collected saliva was measured and expressed in mL/min. Stimulated salivary flow was defined as “reduced” when less than 1 mL/min (21).

-Statistical analysis

The Statistical Package for the Social Sciences (SPSS) version 20.0.0 and the Open.Epi (Dean AG, Sullivan KM, Soe MM. OpenEpi: Open Source Epidemiologic Statistics for Public Health, Version 2.3.1. www.OpenEpi.com, updated on 19/09/2010 and accessed on 04/01/2017) were used for data processing to obtain descriptive statistics and to perform bivariate analyses. SF-36 domains, their summary scores, and the Work Ability Index were considered as the main outcomes; salivary flow, the DMFT index and its components, and periodontal disease were the main predictors. T-tests were used to compare the means of the SF-36 domains and the summaries according to salivary flow strata (normal vs. reduced). T-tests were also used to compare DMFT means according to collapsed categories of the Work Ability Index. Differences between the Work Ability Index category proportions according to salivary flow strata were evaluated by using the chi-square test. Differences in SF-36 means and in Work Ability index proportions stratified according to sex, smoking, drinking, gingivitis, and periodontitis were analyzed by using t-tests and chi-squared testes, respectively. Spearman correlation coefficients (rs) were calculated for data relating the SF-36 indicators with the DMFT index and its components.

-Ethical aspects

The study was approved by the Ethics Review Board (Opinion 711.945 dated 8 July 2014) of the School of Medicine at the Federal University of Bahia, in accordance with Brazilian National Health Council Resolution 466/2012 and the World Medical Association Declaration of Helsinki 2013. All patients signed an informed consent form prior to their inclusion in the study

## Results

-Sociodemographic and clinical data

In the study group of 150 patients with chronic liver diseases, males, African-Brazilians, with low family incomes and low levels of education, predominated. A small proportion of the patients were current drinkers (6.0%) or current smokers (5.3%) ( Table 1). The mean (±SD) age was 51.8 ± 11.1 years.

**Table 1:**
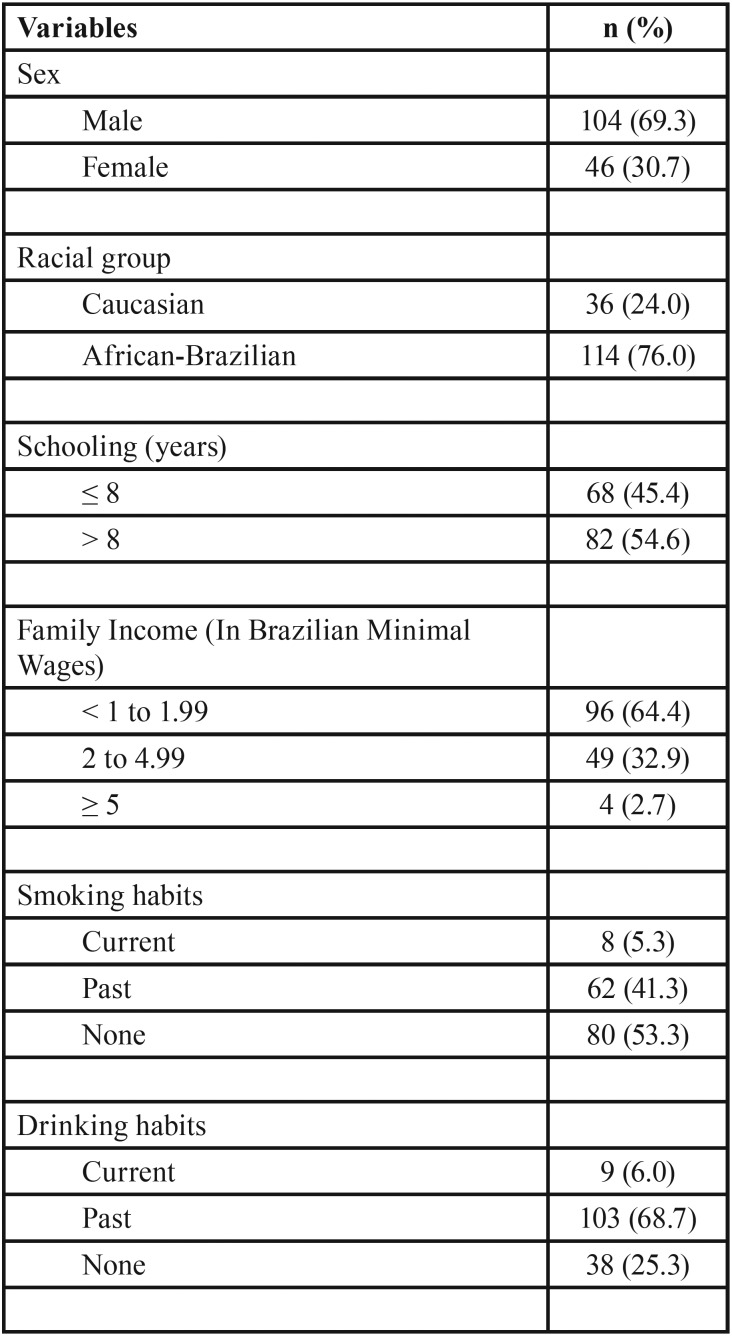
Sociodemographic characteristics and habits of 150 patients with chronic liver disease.

Hepatitis C (50.7%), hepatitis B (16.7%), and alcoholic liver disease (23.3%) were the most common etiologies of chronic liver disease. As comorbidities, type II diabetes was present in 20.0% of the patients, and arterial hypertension in 25.3%. Periodontitis and gingivitis were found in 42.0% and 44.7% of the patients, respectively; 66.0% had reduced salivary flow; and the mean DMFT index was 20.3 ± 8.1. Low work ability was found in 36.7% of the 150 patients. All SF-36 normalised mean scores were systematically below 50 ( Table 2).

**Table 2:**
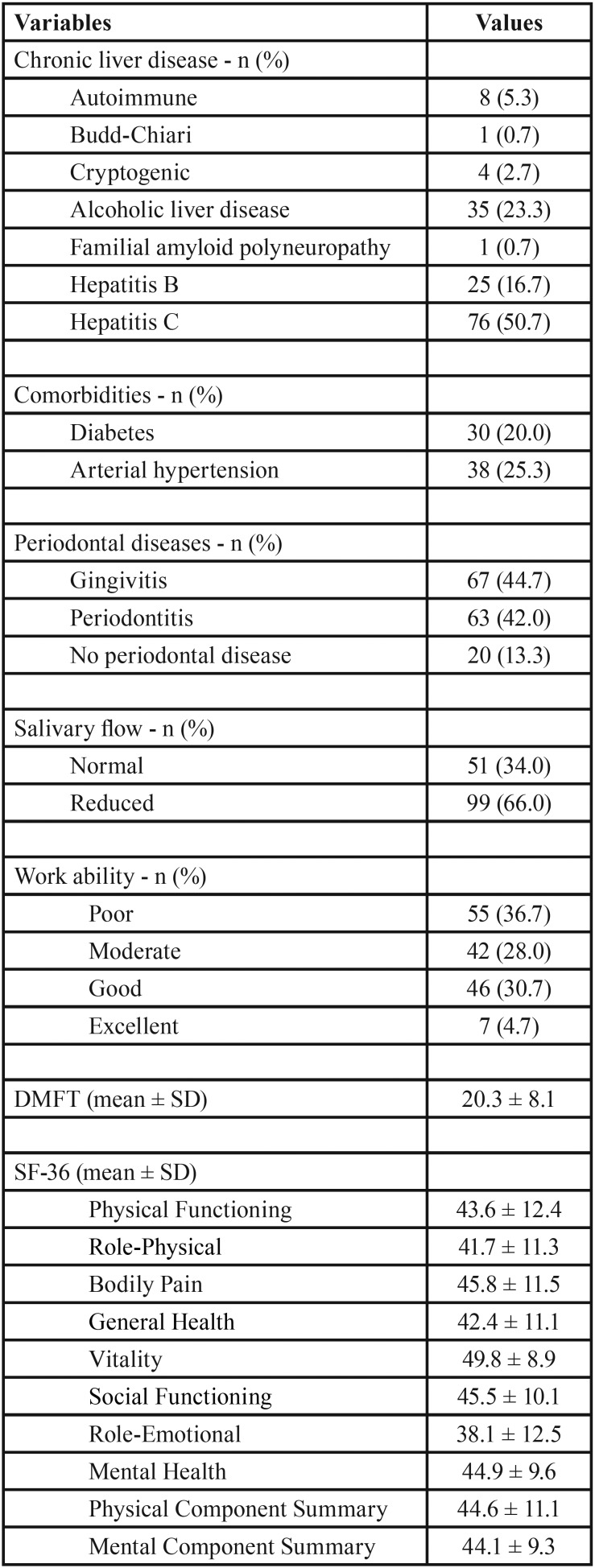
Chronic liver diseases, comorbidities, oral health indices, work ability index and health-related quality of life (SF-36) in 150 patients with chronic liver disease.

-Oral health, health-related quality of life and work ability 

The mean results of all the SF-36 indicators were systematically lower among patients with reduced salivary flow than in those with normal salivary flow. All comparisons showed statistical associations (*P* <0.05 or less), except for the Vitality domain (*P* = 0.110) ( Table 3).

**Table 3:**
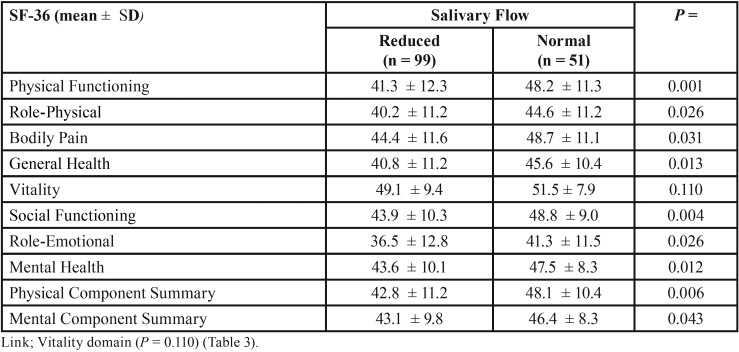
SF-36 normalised domains and summary scores according to salivary flow in 150 patients with chronic liver disease.

The HRQOL scores for Physical Functioning, Role-Physical, and Physical Component Summary were strongly correlated (*P* < 0.005 or less) with the number of Missing Teeth and the DMFT index ( Table 4).

**Table 4:**
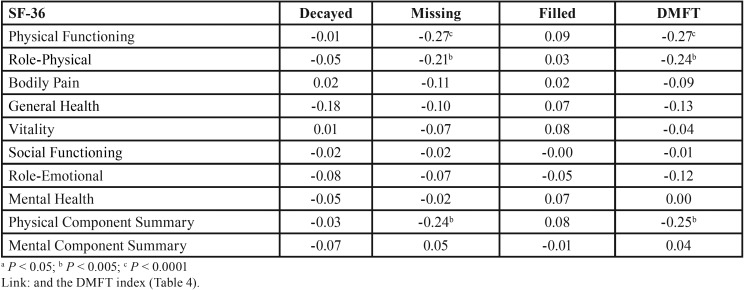
Spearman correlation coefficients between SF-36 domains, summary scores (mean ± SD) and DMFT index components in 150 patients with chronic liver disease.

Reduced salivary flow was more frequent (*P* < 0.004) among patients with poor or moderate work ability than in the set of patients with good or excellent work ability ( Table 5).

**Table 5:**
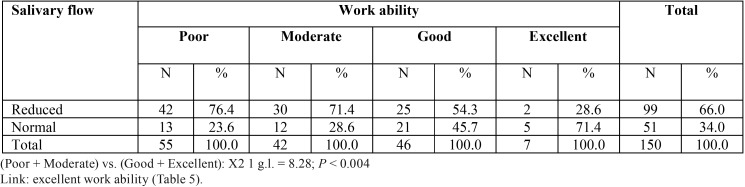
Work ability according to salivary flow in 150 patients with chronic liver disease.

Patients with poor or moderate work ability presented higher (*P* < 0.0001) means of the DMFT index (22.1 ± 8.1) than those with good or excellent work ability (17.1 ± 7.2).

Periodontitis and gingivitis were not significantly associated (*P* > 0.05) with health-related quality of life or with work ability (data not shown).

The means for the SF-36 domains and the component summaries did not show striking differences (*P* > 0.05) according to sex, smoking, drinking, gingivitis or periodontitis. SF-36 domains and its component summaries did not show strong correlations with age, except for Physical Functioning (Spearman correlation coefficient = -0.18; *P* = 0.02). Work Ability Index strata did not vary significantly (*P* > 0.05) according to the same variables (data not shown).

## Discussion

All SF-36 normalised mean scores for the patients with chronic liver disease fell well below 50 and were particularly low for the Role-Emotional domain ( Table 2). Reference values for normalised scores were taken from the general population of the USA (15). Unfortunately, because of a lack of normalisation, we could not make adequate comparisons between the SF-36 scores in our study group and those from studies undertaken in Brazil. Most studies usually report “raw”, non-normalised, scores, therefore impairing meaningful comparisons between studies.

Reduced salivary flow was strongly associated with all the SF-36 indicators, except for Vitality. Sixty-six percent of patients with chronic liver disease in our study presented reduced salivary flow. This can reduce dental remineralization and antimicrobial activity in the mouth. Reduced salivary flow must be considered as an important factor that mediates the relationship between oral health and health-related quality of life in patients with chronic liver disease. Another study reported associations between reduced salivary flow and periodontal disease, caries, and oral mucosal lesions (7).

The mean of DMFT index was quite high in patients with chronic liver disease: 20.3 ± 8.1. Both the DMFT and the number of Missing Teeth showed strong negative correlations with the HRQOL scores for Physical Functioning, Role-Physical, and Physical Component Summary. These indicators of poor oral health may affect certain basic functions, such as the ability to eat, speak, and socialize, impairing the individual’s interpersonal relationships and, consequently, leading to poor health-related quality of life.

Classical epidemiological studies among Finnish workers associated work ability with employee well-being, organizational commitment (22), high productivity and high work quality (23). Some studies have emphasized health as a major determinant of work ability (22,24). Among patients with chronic liver disease, poor work ability was strongly associated with reduced salivary flow ( Table 5) and precarious oral health status, as revealed by higher DMFT indices.

Periodontitis (42.0%) and gingivitis (44.7%) were frequently found in our patients, but these periodontal diseases were not associated with low health-related quality of life or poor work ability. However, previous studies have found associations between periodontal disease and low quality of life (25-29). Most of these studies (25-28), have used the Oral Health Impact profile (OHIP-14) to measure the impact of oral disease on oral health-related quality of life. However, we opted to use the SF-36, a generic instrument that provides physical and mental summary components of the health-related quality of life. The SF-36 has been frequently used to evaluate health-related quality of life, including the oral health of patients with chronic pathologies, like chronic liver diseases (20) e HIV31.

Some study limitations must be addressed. Cross-sectional design studies have inherent methodological limitations, such as the difficulty of establishing the correct temporal sequence between exposure and effect. This is an exploratory study, developed in a single reference centre for chronic liver disease, which implies low external validity.

Small sample size in may lead to type II error. Despite this, this study found systematically lower means for SF-36 scores and poorer work ability among patients with chronic liver disease who presented reduced salivary flow. Small sample size precluded the possibility of performing a thorough evaluation of confounding and effect modification. However, we found that the main dependent variables (health-related quality of life and work ability) were not associated with certain covariables (sex, age, smoking, drinking, gingivitis, and periodontitis).

## Conclusions

This study among patients with chronic liver disease found strong associations between poor oral health (reduced salivary flow or high DMFT index) with low health-related quality of life and with poor work ability. These findings reinforce the need of specialized stomatological care for patients with chronic liver diseases.
